# The Peril of the Pocket-Handkerchief

**Published:** 1921-04-02

**Authors:** 


### The Peril of the Pocket-Handkerchief.

### BY A CORRESPONDENT.

It is a remarkable thing that during recent great offensives of influenza and scarlet fever, little or no attention has been directed to what is probably an important source of infection.

Whatever the germs of influenza and scarlet fever may be, there can be no doubt that they invade and occupy the mucous membranes of the nose and pharynx, and that the secretions and catarrhal discharges of these membranes are infectious and dangerous. Yet the pocket-handkerchief of the patient, which is bound to be more or less smeared and saturated with infectious material from the upper air-passages, is flaunted and flourished in the face of the community, and the public are left quite uninstructed in its inherent dangers. In the organic substance of the ordinary handkerchief, and in the organic catarrhal matter on it, germs can feed, and thrive, and multiply, so long as the handkerchief is moist, while, as soon as it is dry, every flourish shakes millions of them into the air. A dirty handkerchief, used—it may be for some days—by a patient suffering from catarrh of any kind, is covered with decomposing organic matter, and, kept at a steady temperature in a warm pocket, forms an admirable hotbed and incubator for the intensive cultivation of microbes.

It is better, of course, to hold a clean handkerchief before the mouth during coughing fits than to cough at large, but a handkerchief is at best a poor barrier to oppose to a violent cough, and if it be soiled and contaminated will be of little or no use.

The public are told to douche their noses and to gargle their throats. They are told, in cases of influenza, to use masks, which are about as effective as herring-nets to intercept germs, interfere with free breathing, and render the air inspired unhealthily warm and wet. They are told to avoid crowds, which are probably unavoidable; they are told to sterilise eating utensils; and they are given much other advice, and yet nothing is said of that little flag of Death, the pocket-handkerchief, and it goes on spreading disease from hand to mouth and from laundry to living-room. From laundry to living-room, we say, for few measures are taken to disinfect contaminated handkerchiefs and to prevent the infection of other handkerchiefs in the wash. During the great influenza epidemic laundry-workers were specially attacked by the disease, and no wonder.

If infectious catarrhal diseases are to be checked, the handkerchief as a source of infection must be faithfully dealt with. At sanatoria handkerchiefs used by consumptive patients are always soaked in antiseptic before being washed, and all handkerchiefs should be treated in that way. Further, the public must be taught that handkerchiefs, like bandages, must be changed as soon as they are smeared, and that a dirty handkerchief in the pocket is a source of danger both to its owner and to the community. It must be remembered, too, that under certain conditions the pocket itself is liable to become septic and dangerous. The writer has demonstrated tubercle bacilli in the handkerchief-pockets of consumptives. Probably in times of epidemic and pandemic it would be a good thing to sprinkle both handkerchief and pocket with some volatile disinfectant, in addition to taking other more thorough disinfectant measures. It would be a good thing, too, if people could be persuaded to use pocket-handkerchiefs and to burn them as soon as soiled.

Whatever measures be taken, it is necessary that more attention should be paid to handkerchiefs as sources of infection.

